# A mixed-method evaluation of the impact of introducing 52 digital X-ray systems in Ghanaian health facilities on tuberculosis case finding and quality of care

**DOI:** 10.1371/journal.pgph.0006874

**Published:** 2026-07-21

**Authors:** Mirjam I. Bakker, John K. Krugu, Christina Mergenthaler, Yaw Adusi-Poku, Rita Patricia Frimpong Amenyo, Bernard Wadie Adu, Felix Kwami Afutu, Mawuko Mensah, Zeleke Alebachew, Robert Asambobillah, Benjamin Apam, Chantale Lakis, Maurits Verhagen, Nwanneka Okere

**Affiliations:** 1 KIT Royal Tropical Institute, Global Health, Amsterdam, The Netherlands; 2 Design Health Consult Ltd, Accra, Ghana; 3 National TB Program, Ministry of Health, Accra, Ghana; 4 Delft Imaging, ‘s Hertogenbosch, The Netherlands; University of California Irvine, UNITED STATES OF AMERICA

## Abstract

Between end 2016 and early 2018, 52 digital X-ray systems were installed in public health facilities across Ghana under the ORIO program, funded by the Dutch Ministry of Foreign Affairs and the Government of Ghana. The project aimed to detect more people with TB by expanding access to digital radiology, supported by training, maintenance, and project management. This evaluation assessed the project’s contribution to TB case finding, diagnostic capacity and access to TB diagnostic services between 2017 and 2022. The evaluation used a mixed-methods approach, including 21 key informants interviews from national to facility level, and 87 semi-structured interviews with TB coordinators, OPD doctors, radiographers and X-ray clients at 26 facilities. We analyzed district- and facility-level TB notification data (2014 – 2022), and numbers of X-rays and chest X-rays performed. The project was widely perceived to have improved access to TB screening and diagnosis by reducing cost and distance barriers. Challenges included a shortage of radiographers, lack of uniform screening guidelines, and poor internet connectivity. While TB notifications increased at district level after the installation of the digital x-ray machines, increases were not seen at facility level, suggesting machines served a regional diagnostic function. Interviews supported this interpretation. Considerable variation in TB notifications trends across sites was observed. Underutilization and a low proportion of chest X-rays likely played a role. Machines were more often used for non-chest imaging. The project strengthened the health system through increased access to X-ray screening and diagnostic services for TB, lung-related diseases, and other conditions. Effective and sustainable integration of digital X-ray technology requires understanding of the operational context, robust protocols, and addressing staff shortages and turnover. Reliable internet is critical for real-time monitoring and optimizing machine usage to improve TB management and expand diagnostic capabilities.

## Background

Tuberculosis (TB) remains one of the most frequent causes of death among adults globally, despite being curable. In 2023, TB was most likely the leading cause of death again, ranking above COVID-19 and HIV/AIDS [1]. Globally, an estimated 2.7 million people with TB were not notified in 2023 [1], of which many did not access care, did not get diagnosed or were not reported. In Ghana, only 44% of the estimated 44,000 new TB patients were notified in 2023 [2]. To close this gap more people at risk for TB need to have access to care, be screened for and diagnosed with TB, and subsequently treated and notified. However, studies have shown that one of the problems in Ghana is poor access to TB diagnostics [3].

The nationwide TB prevalence survey of 2013 revealed not only a much higher TB burden than previously estimated, but also a high proportion of new TB patients who were smear-negative (68%) and a significant proportion (59%) who did not report signs or symptoms of TB during screening [4]. This urged the National TB Control Programme (NTP) of Ghana to undertake more actions to find the undiagnosed (‘missed’) people with TB and to revise the screening and diagnostic algorithms to create a more sensitive process which will better identify the large number of people with TB who are asymptomatic. The 2015–2020 Ghana Health Sector TB Strategic Plan set the country’s program priorities for early screening, detection and enrolment in TB treatment [5]. Chest X-ray as an initial screening tool has very high sensitivity [6] and has a high potential for closing the treatment gap. Studies have shown that adding chest X-ray as an additional screening tool increases the yield of intensified case finding at health facilities [7–9].

In 2009, the Ministry of Health of Ghana realized the need to expand access to digital Chest X-ray as a screening and diagnostic tool and subsequently requested a grant and bank loan from the ORIO program of the Dutch Ministry of Foreign Affairs to install over 50 digital Chest X-ray machines across Ghana with supporting infrastructure and training to improve TB case detection [10]. The ORIO program, initially managed by The Netherlands Enterprise Agency (RVO) and later by Invest International, supported investments in public infrastructure in low and middle-income countries [11]. Implementation in Ghana began in 2016 [12], by which time the TB diagnostic landscape had changed; WHO endorsed GeneXpert in 2011 as initial test for people presumptive of drug-resistant TB, people living with HIV (PLVIH), and children, and expanded its use to all people with presumptive TB in 2013 [13]. In Ghana, GeneXpert coverage increased rapidly, from fewer than 20 machines in 2015 [5] to becoming the primary diagnostic TB test in 2017, replacing microscopy. In 2021, WHO also endorsed computer-aided detection for interpreting digital chest X-rays for TB screening and triage [14]. Within this evolving landscape, Ghana’s NTP initially planned to use digital X-ray for intensified case finding to prioritize patients for microscopy and later GeneXpert, while also enabling TB diagnosis in bacteriologically negative patients.

This paper describes evaluation findings of the study on the impact of the installed digital X-ray equipment and training as part of the ORIO project on TB case detection, diagnostic capacity, and access to TB diagnostic services between 2016 and 2022. It also considered improvements in the health system in general, considering the perspectives of management, healthcare workers, and patients.

## Methods

### Ethics statement

We received ethical approval for this study from the Ghana Health Service Ethics Review Committee on September 1, 2022 (GHS-ERC Number: 029/08/22). We also asked and received administrative permission from the District Directors of Health Services and the Heads of the health facilities where data was collected. Written Informed Consent was obtained from all respondents. All respondents were assured of confidentiality.

### Study design

The evaluation followed a mixed-methods approach entailing quantitative secondary data analysis of both routine surveillance TB data from the NTP and the project data, qualitative key-informant interviews, semi-structured interviews, observations and a desk review of relevant project documents. We used a blended Donabedian–Levesque framework to structure our evaluation [15,16]. Data collection was done between October and December 2022. Surveillance data collected spanned the period before and after the X-ray installations (2014–2022).

### The intervention

The project ‘ORIO 09GH05 Accelerating TB Case Detection in Ghana’ aimed to detect more (pulmonary) TB patients by expanding access to digital chest radiology for patients visiting public sector facilities. Implementation commenced in February 2016, after which 52 multi-purpose digital X-ray systems (Delft EasyDR) were installed with CAD4TB software version 6, which was replaced with version 7 in 2021: 48 in hospitals (including district, regional, secondary and tertiary hospitals) across the country (19 fixed stations and 29 containerized), two in specially outfitted vans and two portable X-ray systems in transport cases stationed with the NTP. Sites were selected based on needs assessment including criteria such as the lack of functional X-ray equipment, high TB notification and daily patient throughput, and viability of replacing the functional analogue X-ray system in a high throughput setting. Furthermore, 16 radiological viewing stations were provided in regional hospitals and NTP headquarters to enable teleradiology services. The installation of equipment was accompanied by system-related (engineers, radiographers, IT staff) and programmatic training (clinicians, radiologists), maintenance (preventive and corrective), project management and a truck plus some 4-wheel drive vehicles. The project ended in February 2025.

### Study sites

**Fig 1:**
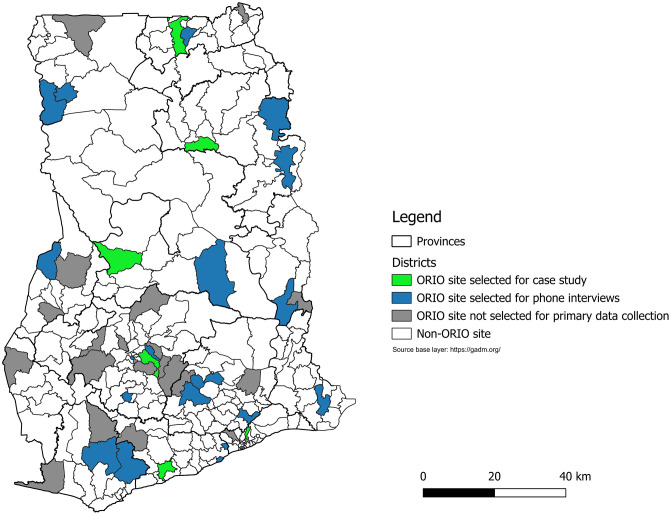
Map of Ghana showing the six districts in which the case study sites are located (green), the 20 districts in which the phone sites are located (blue) and the other districts with an ORIO site (grey). Administrative boundaries shapefile from https://gadm.org/download_country.html.

Primary data collection for the evaluation was done face-to-face at six selected case study sites where the digital X-ray systems were installed, and through phone interviews at an additional 20 sites (Fig 1). The criteria for selecting case study and phone interview sites aimed to represent the different types of X-ray systems installed (container or fixed installation), facility location, i.e., urban or rural, and facility type (district, regional, secondary or tertiary). The six case study sites were selected using a stratified multistage sampling approach. Ghana’s three main ecological zones served as first-stage strata, regions as second stage, and health facilities served as primary sampling unit. Two regions were randomly selected from each of the three ecological zones. Within each region, one facility that received an X-ray system was purposively selected based on the criteria above. The 20 facilities for phone interviews were selected to ensure representation of all regions that received ORIO digital X-ray systems in the final sample proportional to the number of systems received, in addition to meeting the criteria above. For the secondary data analysis, data from all 48 facilities with an ORIO digital X-ray system and their respective district (n = 46) were included.

### Primary data collection and analysis

Twenty-one key informants were interviewed, 13 were face-to-face, 6 via telephone and two of which gave written responses. Synthesized Narrative Exploration (SNE), an in-depth interviewing method that entails the further exploration of summarized findings from the desk review to build on “what is already known”, was employed. Key informants included stakeholders in The Netherlands (4), national level in Ghana (5), regional managers (3), hospital in-charges and TB coordinators at the facility or district level (9).

A total of 87 semi-structured interviews (SSI) were conducted using a digital data collection tool on tablets, 45 were done face-to-face, and 42 were conducted via phone; with 57 healthcare workers (HCWs) and 30 patients. The selection criteria for HCWs included the ability to refer patients for X-ray, being the TB focal point of the facility, or being the person operating the digital X-ray machine, with the added requirement of being available on the visit day or at least having a reachable phone number. Twenty-one radiographers/X-ray operators, 18 TB coordinators/DOT nurses and 18 doctors were interviewed, of which 90%, 67%, and 83% were males, respectively. Five patients from each case study site who had used the X-ray systems on the visit day (exit interviews) or at least within the last month for TB screening or TB diagnostic purposes (selected from registers), and who were adults were conveniently selected and interviewed at the facility, at their homes or through phone calls. We aimed to conduct 2–3 exit interviews per site. This number increased where no TB related clients were identified. Of all patients who had cough (11/30), 82% were males, and 72% had an X-ray done within the same week of the interview. Additionally, the case study sites the X-ray system was observed using a digital checklist. All tools were piloted in non-selected facilities with a digital X-ray system and adjustments to the tools were made where necessary.

The key-informant interviews were recorded and transcribed. SSI tools for HCWs and radiographers were slightly revised after being used in the case study sites. A thematic analysis was conducted using the key informant interviews. The analysis used both deductive and inductive approaches. A codebook was used to guide the coding process, which facilitated the exploration of predetermined themes, while the inductive approach enabled the identification of new themes. The results of the semi-structured interviews were tabulated and presented for subgroups where relevant. For the characteristics of the patients and HCWs interviewed we refer to S1 Table and S2 Table.

### Secondary data analysis

To study the quarterly TB notifications at facility and district levels before and after the installation of the ORIO digital X-ray systems, we combined subnational aggregated data from NTP routine data report from 2014-2017 with data from the District Health Information Management System 2 (DHIMS2) for the years 2018–2022. These two sources were matched at the facility- and district-level. Facility notifications for 45 out of 48 facilities could be matched in both databases. The remaining three facilities had incomplete data for the 2014–2022 period. District-level data were more challenging to match due to the re-districting of 216–261 districts in recent years: 40 districts were matched and used in analysis. At the facility and district levels, we calculated the expected number of bacteriologically confirmed (B+) and all forms (AF) TB notifications during implementation, based on the linear trend of 12 quarters of data prior to the quarter in which the X-ray systems were accepted. We selected a linear trend as it is both a standardized and pragmatic method for measuring ‘additional’ notifications, as well as being broadly interpretable [17]. These expected notifications served as baseline (N_exp). The additional number of B+ and AF TB notifications was calculated as: ΔN = N_obs − N_exp, where N_obs is the observed notifications during the quarters following the acceptance date. The percentage change from baseline was calculated as (ΔN/ N_exp) × 100. We also calculated the proportion of TB cases B+ out of all patients notified for both baseline and intervention periods.

To assess whether B+ and AF TB notifications increased during program implementation at district level, we used a generalized linear model (GLM) with a Poisson distribution and log link. The model estimated changes in district notification counts over time, including terms for the pre-intervention trend, immediate level change at intervention, and post-intervention trend. Robust standard errors accounted for potential autocorrelation and overdispersion. A negative binomial model was fitted as a sensitivity analysis; as the estimated dispersion parameter was near zero, the Poisson model was considered appropriate.

DHIMS2 also provided annual TB care cascade data from the screening activities for all DHIMS2 reporting sites for the years 2018–2022, which we used to calculated the TB cascade indicators annually from 2018 through 2022, proportion of OPD attendees screened for TB, proportion of people screened for TB found presumptive for TB, proportion of people presumptive for TB who were tested for TB and proportion of people tested for TB who were diagnosed with B + TB.

To understand the utilization of the digital X-ray machines, we relied on 1) Delft Imaging maintenance reports providing the number of X-rays performed per ORIO site disaggregated per maintenance cycle from 2016 – 2022, with the first maintenance cycle done early 2018 and 2) CXR Images Reports of the ORIO project which provided quarterly number of X-rays and chest X-rays taken per hospital for the years 2018–2021, only including images uploaded to the National TB Program archive X-ray 2016 – 2022. The number of X-rays, as well as the number and proportion of which were chest X-rays, were calculated and plotted annually from 2018 to 2022. As the maintenance reports gave a more complete picture across the facilities, we applied the percentage of chest X-rays from the national archive to this source to estimate the number of chest X-rays done. To understand the number of trainings performed and number/types of participants, we relied on the ORIO training reports produced between 2016 and 2022, and yearly maintenance reports since 2018. All data were analyzed in Excel and Stata Version 15 SE.

## Results

### Structure

#### Input: Equipment installation, training, operational status of machines and access.

The machines were installed between Nov 2016 and Feb 2018. Most of the sampled X-ray systems (23/26–88%) were in use during the study. Besides not having an operator (2), non-functionality was caused by being temporarily out of order (1). Most (17/21) systems evaluated did not operate on a 24/7 basis. The assessment revealed an initial shortage of operators for the X-ray systems sponsored by a national law requiring the machines to be operated only by radiographers. A consensus reached with the Ghana Society of Radiographers to recruit and train biomedical engineers and medical physicists as operators improved the situation in the short run, while recruiting more radiographers into public service remained the goal. Of the facilities 70% (14/20) had a radiographer who had received at least one (refresher) training under the project, or a technician trained on-the-job. Retention remained a problem however, especially in the rural areas as alluded by this regional manager: *“I mean personnel who have been there for let’s say a year. Before then, the place was being manned by service personnel from other places. So the person will come, a few months he leaves, so staff attrition too has been something that has worried us.”*

The project supported the training of 759 healthcare workers in total. Those who received at least one form of training were 223 Doctors, 195 Radiographers, 114 Physician assistants, 77 Equipment Engineers, 58 IT managers, 43 Biomedical Engineers, and 14 Radiologists, among others.

Most patients (21/30) agreed that access and quality of X-ray services had improved in their community over the past 5 years. Reasons provided were improved availability of X-ray services, leading to less referral, and a quicker, more comfortable and accurate process. Of the patients interviewed 65% had less than 30 minutes or 5 kilometers of travel, still some had to travel far to reach the X-ray facility; 57% sought care directly in the facility with the X-ray, 30% were referred from another facility and 13% changed facility without referral (on their own choice). Most patients (74%) indicated that they received same-day X-ray results. Although X-ray services to screen for TB should be free of any charges, 6 of 12 patients with a cough or on TB treatment indicated they had to pay for the X-ray services. The median payment for X-ray services (n = 23) was Ghc50; some indicated they only had to pay Ghc20 for printing the X-ray, and others paid up to Ghc100. Out of 30 patients, four (13%) mentioned financial costs as a barrier to accessing the services in their community. Most (97%) of the patients were satisfied with the timeliness of communication and services, while only 55% felt adequately informed about costs in a timely manner.

#### Internet availability/tele-radiology dream.

The infrastructure for internet connectivity was still developing and epileptic at the time of the project implementation, and the X-ray machines were primarily operated offline. Though some regional hospitals were designated for viewing and interpreting X-ray slides from peripheral health facilities, there was no evidence that the teleradiology component of the project was ever operational. The major reason given that limited the full digital experience planned for the project, was the uncompensated increased workload of designated radiologists, besides non-payment of internet bundle by the NTP and poor internet connectivity.

#### Availability of digital diagnosis.

Screening and diagnostic options available in health facilities for the diagnosis of TB, lung-related diseases, and other conditions, such as bone and joint X-ray, increased due to the availability of the installed X-ray machines. Another cited contribution was the faster turnaround time for getting X-ray results, which expedited the diagnostic process.

“*Now, let’s take TB too for example, you suspect a case of TB in a patient and … request for the imaging. And of course this patient is going to go by public transport (to other hospitals having an X-ray machine), she will come back with X-ray couple of days or weeks later. God knows the number of people she contacted. The patient may not even come back, you have to follow up and stuff. But now the machine is here, you get a client, then follow-up is so erhh…is regular…in short right now we are comprehensively managing these cases, …its not bit-by-bit bit-by-bit. So it is actually a game changer”. -* Facility level respondent

#### Capacity to diagnose TB – personnel.

Respondents indicated that the availability of X-ray machines broadened diagnostic options and may have supported more timely TB case finding including extrapulmonary TB across different patient groups.

*“…but ever since the machine has come, you can realize that there are some cases we able to get those who are actually positive, who are, what’s the right word, I want to use the right word, who are extra pulmonary, exactly, who are extra pulmonary, our extra pulmonary cases has increased, our case [detection]. It’s quicker for us to diagnosis somebody” -* Regional level respondent

The additional installation of CAD4TB in the machines empowered healthcare workers without specialized training to use AI technology to identify people with presumptive TB, thus increasing the screening capacity for TB.

#### Maintenance.

Delft Imaging made yearly preventive maintenance visits to all sites since 2018 and continued till the end of the project in February 2025. Regional equipment managers, IT officers and biomedical engineers from the GHS have participated in multiple training activities and maintenance visits, which positions the GHS to sustain maintenance of the X-ray systems after February 2025.

### Process

#### Screening and diagnostic processes.

Intensified TB case finding (ICF) was originally planned for the project using X-ray as a screening tool for all people with presumptive TB (after verbal screening). Though a national ICF screening algorithm was developed, its dissemination and implementation across facilities remained limited. There was, therefore, minimal evidence of a standard algorithm guiding TB screening activities as different algorithms were found in use across facilities. For example, only 38% of TB focal persons interviewed reported using the NTP algorithm to diagnose TB, while the rest used either facility specific, CDC, or WHO algorithms. Other facility staff were generally not familiar with TB screening algorithms. Relatedly, many HCWs (40%) were unsure about the CAD4TB threshold to classify an X-ray as abnormal. Respondents gave scores ranging from 40-95. Likewise, patient pathways to further care after accessing the X-ray varied across facilities from starting TB treatment, request for Xpert testing, testing for co-infections, or simply referral to the TB unit. Clear evidence that the project led to increased ICF is lacking, as there was no change in the screening or presumptive rate, aside from exceptions at individual facilities.

#### M&E processes.

There was no evidence of any system in place to collate and link clients’ ICF screening data to their X-ray results even in facilities where an electronic health information system (eHIS) existed. Therefore, the summary of all X-rays done by the machines was available but not linked to other client data such as client demographics, TB screening, and other lab results. Consequently, further analysis with the X-ray data to generate evidence for optimizing the use of the machines was difficult. Also, the paper-based data management system used in 86% of facilities, and the lack of a structured reporting system compounded the difficulty of monitoring the use of the machines across facilities.

#### Outputs: Machine utilization.

**Fig 2:**
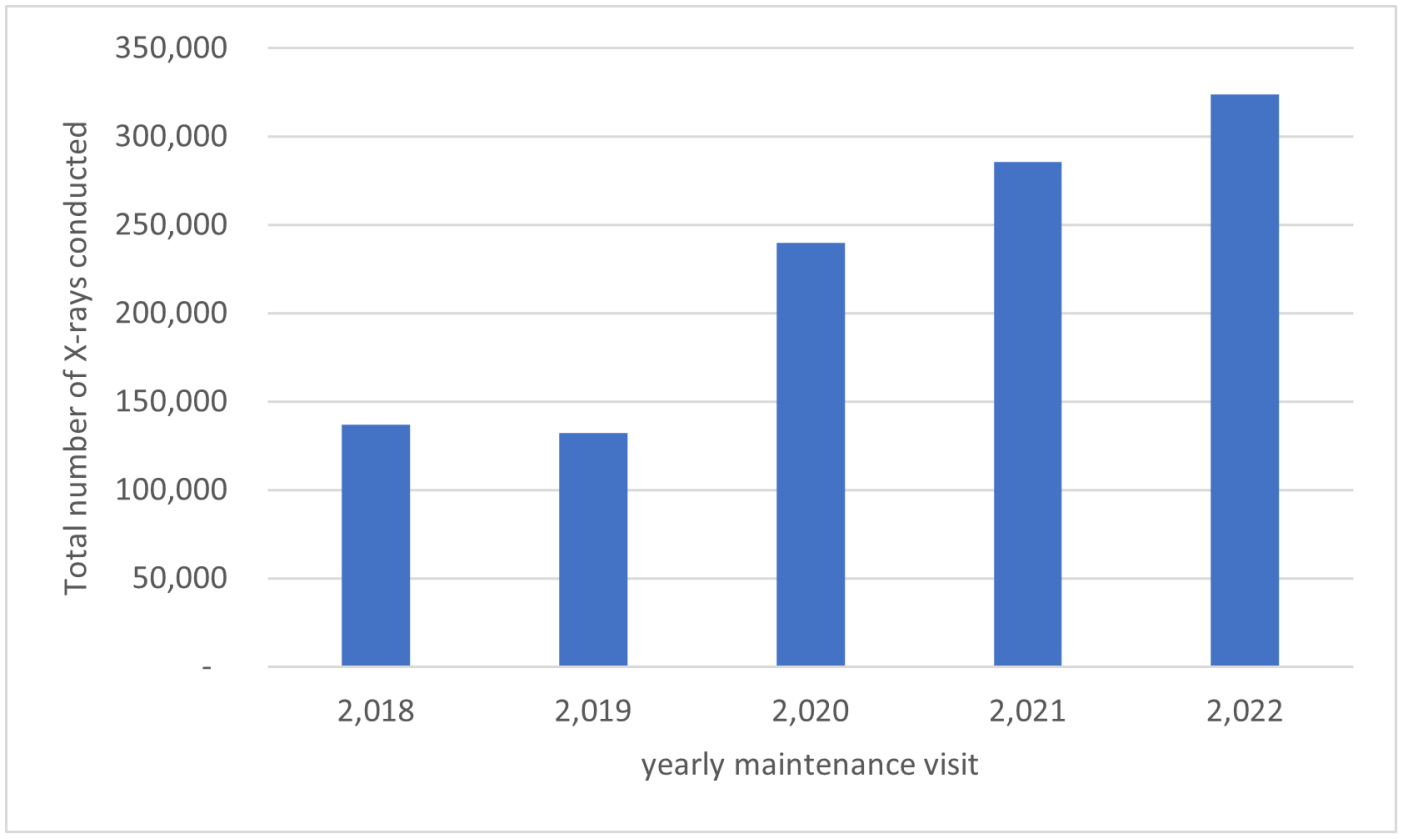
Total number of X-rays conducted across 50 machines (2018-2022) as reported during the yearly maintenance visits by Delft Imaging engineers.

Fig 2 shows an increasing trend in the total number of images taken by the X-ray systems per year up to the last preventive maintenance visits in 2022, totalling 1,119,175 (note: these do not represent calendar years). At the machine level across facilities, utilization data revealed a wide variation ranging from 1,844–105,136 images taken over the review period (2018–2022). The two machines placed in mobile vans, used for outreach TB-screening, contributed to many chest X-rays taken (145,144 and 163,677). In total, 1,065,902 X-rays were shared by facilities to the national archive. In 2016 and 2017, one and 10 facilities respectively shared x-rays; from 2018 to 2021, this number fluctuated between 43 and 49 facilities, and in 2022, only 22 facilities reported X-ray data. Out of all X-rays shared with the national archive, 264,527 (24.8%) were chest X-rays. This proportion fluctuated around 23.6% and 24.1% in 2018 and 2019, dipped to 19.9% in 2020, and rebounded up to 32.7% in 2021, and 27.9% in 2022 although there are fewer data points (facilities) for 2022. The lower percentage in 2020 was explained by the COVID-19 pandemic preventing the two vans with chest X-ray machines going out.

### Outcome

#### TB notifications.

**Fig 3:**
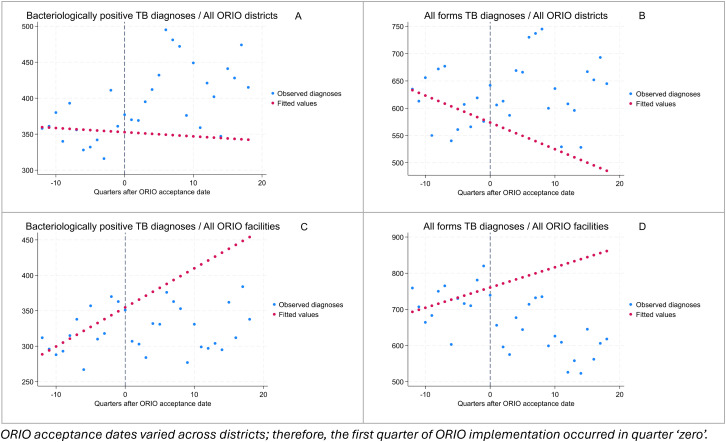
District-level (a and b) and facility-level (c and d) B+ (a and c) or AF of TB (b and d) observed notifications (2014-2022) before and after the acceptance date of the digital X-ray system, and the trend line based on the 12 quarters before the acceptance date.

Fig 3a and 3b show an increase in both bacteriologically positive TB patients as well as all forms of TB patients compared to the expected number of notifications based on the baseline trend after the installment of the digital X-ray systems at the district level, 19.9% increase for bacteriological positive patients and a 20.7% increase in all forms TB patients. This increase was however not seen at the facility-level (Fig 3c and 3d): the number of bacteriologically positive patients decreased by 23.9%, and all forms of TB patients decreased by 22.5% after the installment compared to the expected number of notifications. The slopes of the district-level trendlines (negative) are very different compared to the slopes of the facility-level trend lines (positive); this influenced the ability to reach a positive change compared to the baseline trend at facility level.

The GLM showed a downward trend in all forms TB district notifications during the pre-intervention period (p = 0.021), corresponding to a 0.81% decrease in notifications per quarter (95% confidence interval [CI]: -1.5 - -0.12%). At the time of intervention, there was a statistically significant immediate increase in notifications (p = 0.009), reflecting an 11.6% relative increase (95% CI: 2.8-21%). Although the slope of the post-intervention trend was close to zero (-0.1%), it did not significantly differ from the pre-intervention trend (p = 0.123). For B+ district TB notifications the negative baseline trend was not significant, with a marginally significant (p = 0.058) 13.4% relative increase at start of intervention (95% CI: -0.4-29%), and while the post-intervention trend was positive it did not significantly differ from the pre-intervention trend.

At the district level, the proportion of TB patients with bacteriological confirmation out of all TB patients diagnosed after the digital X-ray systems were installed was 66.3% compared 58.1% during the baseline period. At the facility level, the proportion of TB patients with bacteriological confirmation during the intervention period was 52.1%, which had also increased by 8 percentage points compared to the baseline (44.0%).

Substantial variation was seen in the percentage change compared to expected between the individual facilities and districts. Of the 45 facilities included in the analysis, 18 showed ≥20% lower AF TB notifications than expected, while 17 facilities showed ≥20% higher AF TB notifications.

**Fig 4:**
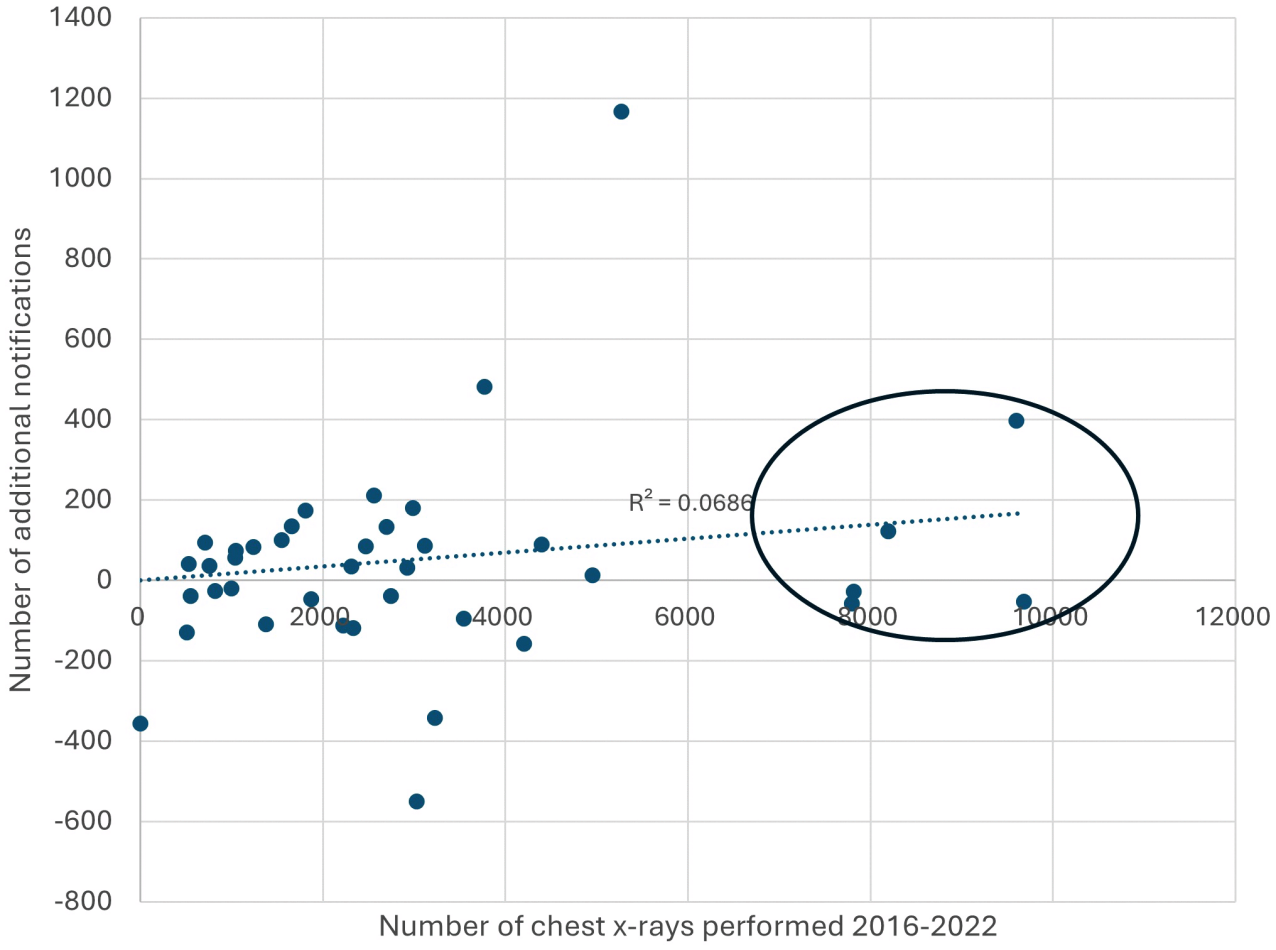
Relation between the number of chest X-rays performed from installment till 2022 and additional AF TB notifications at district level (note: chest X-rays at Legon hospital are not included).

Fig 4 shows the relation between the usage of the machines for chest X-rays and the additional number of TB notifications at the district level with a fitted linear trend line. Each dot represents one district. There is a slight positive trend, but with poor fit likely due to some outliers. Clearly, some districts performed many chest X-rays (those within the circle in Fig 4), but did not show an increase in TB notifications. However, it also shows that to make a substantial impact on notifications, a substantial number of chest X-rays have to be performed.

#### Quality of TB and non-TB diagnosis.

HCW facility respondents corroborated the positive impact of the installed X-ray machines on the screening and diagnosis of TB by mentioning the following advantages of X-ray in TB detection: more accurate clinical diagnosis for those that cannot produce sputum, or when GeneXpert is negative or not (yet) available; X-ray results provide a clear distinction between pneumonia and TB; it can be used as a screening tool among OPD patients and PLHIV; and it is easy and fast. The respondents also noted that the availability of X-ray machines expanded diagnostic options, potentially contributing to improved diagnostic assessment and overall quality of care.

## Discussion

Our evaluation found that installing digital X-ray systems across Ghana affected the health system diversely, for example improving diagnostic capacity, quality, and access, for TB and other conditions for which an X-ray is a screening or diagnostic tool. The project increased the potential for teleradiology and real-time monitoring of TB case detection, though these have not yet been successfully integrated into routine health system processes. Overall TB case notification improved at the district level during the intervention, though wide variation was observed between districts/facilities, which could partly be explained by the intensity of the use of the digital X-ray machines. In Ghana, TB case notification is done at the facility where treatment is initiated, not necessarily at the facility where the diagnosis is made. Therefore, most of patients diagnosed at the district hospital with the digital X-ray likely started treatment and were notified at a lower-level facility. This may explain the increase in TB notifications observed at the district level, in contrast to the facility level; however, the degree to which this increase is attributable to the intervention remains uncertain. Completeness of data entry and capacity to quality assure data often vary across facilities, which may help to explain some of the counterintuitive differences between district and facility level notification trends. The GLM confirmed the pre-intervention decline in all-forms TB notifications at district level and showed a significant immediate increase at intervention onset; the near-flat post-intervention trend (though not significantly different from the pre-intervention trend) suggests that the immediate increase observed at onset was sustained over time; however in the absence of a control group, it is not possible to disentangle the intervention effect from broader secular trends or external influences.

A good understanding of the Ghanaian context and the requirements of an effective digital health intervention during the implementation period provides an important basis for interpreting the results of our evaluation. For example, government buy-in is a crucial factor influencing the adoption, stewardship, organization, technical and financial support necessary for the successful implementation of such a digital intervention [18]. Despite the high-level stakeholder engagement that occurred during implementation, evidence from similar efforts at integrating digital health tools into health systems shows that sustainability is not guaranteed, underscoring the complexities involved in sustaining such interventions [19].

An initial bottleneck recognized and mitigated during project commencement was the limited availability and skewed distribution of radiographers in the country towards urban regions [20,21]. The short-term resolution to task share by training other health workers like physicists and biomedical engineers to operate the X-ray machines while increasing the enrolment and training of radiographers is a strategy that has been employed in other public health programs to improve access to care, e.g., hypertension [22,23], surgeries [24], etc. While teleradiology has helped to mitigate the limited availability of radiologists for X-ray film interpretation in other settings [25], as was shown in Mali [26], our evaluation did not show the same promising results. In contrast, our findings suggest that the teleradiology component could not take off due to the underdeveloped digital infrastructure, and a severe shortage of radiologists managing uncompensated extra workload and limited familiarity with teleradiology [18,27,28]. Incentives and inclusion in coverage policies where health insurance exists, are some strategies proposed to encourage the adoption of teleradiology services [29]. Adequate training is recognized as crucial for the adoption of new technologies [30]; however, the practice of transferring health workers across facilities, as found in our evaluation, disrupts utilization thereby hindering effective service delivery [31].

Due to the versatile role which X-ray systems can play in TB diagnosis, it is important to have clear algorithms to guide their use under diverse scenarios. Procedures for using the X-ray machines in Ghana remain unclear. It varies from using it as an initial screening tool in ICF efforts at the facilities, to facilitating patient screening for more targeted use of GeneXpert machines (as was initially intended), to aiding in the diagnosis of more non-specific presentations of TB [32]. This implies that the proportion of bacteriological positive TB cases out of all diagnosed TB patients could either increase or decrease depending on the efficiency of screening or diagnosis with the X-ray machine. Overall, we noticed a slight increase in the proportion during the intervention.

Findings about trainings conducted during ORIO support the foregoing argument about the versatile use of the machines by showing a high number of training topics targeting a wide range of providers involved in the TB programme. Despite this, the minimal effect observed may be associated with systemic barriers including increased workloads, unclear responsibility and incentives, coupled with unstructured mechanisms to feedback the effect of implementation among stakeholders [33].

Our findings indicate that gaps in the dissemination and implementation of TB diagnostic and TB ICF guidelines may have hindered the effective use of X-ray-supported ICF [34]. Health system factors related to limited human and financial resources, and weak sub-national engagement have been cited as key factors hindering effective TB program coordination and implementation [35]. The resulting variability observed in the screening through the treatment cascade data across facilities and districts aligns with evidence highlighting that poor dissemination and implementation of TB guidelines that characterize clinics in many LMICs settings, constitute a barrier to the implementation of TB guidelines [34,36].

Patient-level data not linked with the X-ray data, as found in Ghana, is a reflection of the predominantly paper-based system for recording TB data during the intervention, which is characterized by being fragmented and non-integrated [37]. Even in the few facilities with an eHIS, the digital machines were operated as a parallel system and not integrated into the eHIS. This is a known challenge and supports evidence that integrating data from a growing number of digital tools, e.g., X-ray machines into the national health information system is critical for enabling real-time access to high-quality, actionable data for disease surveillance, diagnostic performance monitoring, and improved patient care [38]. Though considered promising, operational challenges related to unstable power supply, trained personnel and limited internet and interoperability infrastructure still need to be considered to reap optimal benefits from eHIS [39,40].

The positive relation between the usage of the machines for chest X-rays and the observed additional TB notifications during the intervention was not as strong as expected. Clearly other factors play a role when it comes to number of TB patients being notified, which could be external factors like the roll-out of Xpert testing, and challenges in the TB care cascade. Additionally, factors such as the sequential placement of the X-ray in the screening and diagnostic algorithm and the TB burden among those targeted for X-ray may have also influenced the number of TB notifications.

There is limited clarity in the literature and WHO recommendations regarding the optimal role of X-ray in health facilities as part of ICF strategies to improve TB case detection in high-burden settings. WHO does not recommend systematic screening in all facility attendees but recognizes its value in high-prevalence settings among people with a risk factor for TB [14]. This approach is more cost-effective and feasible when primarily offered to individuals with symptoms or those at higher risk for TB [41]; however, this strategy forfeits the advantage of identifying asymptomatic patients [42]. Follow-up testing with a molecular WHO-recommended rapid diagnostic testing (mWRT) such as GeneXpert is essential, as well as the diagnosis of patients that cannot produce sputum [43]. It is important to closely monitor both machine capacity to process all sputum samples, and skills to diagnose TB, also clinically. Prioritization is key, and digital chest X-ray can support this process [43]. Pooling samples could help reduce the number of GeneXpert tests required [44]. Further implementation research is needed to determine the most effective, efficient, and context-appropriate strategies for integrating screening among health facility attendees to maximize TB case detection.

The integration of digital X-ray systems into the TB diagnostic process, including those fitted with computer-aided diagnosis (CAD) programs, is increasingly adopted by TB programs and has shown promising results in detecting both TB [45] and non-TB abnormalities [46,47]. While there is evidence that combining clinical information with digital diagnostic tools leads to improved TB case detection, the need for more studies estimating population-specific thresholds stratified according to patients’ characteristics is generally advocated [42,45].

A few limitations should be considered in interpreting the findings of this evaluation. First, we used a linear trend of 12 quarters of baseline data from 2014-2017 (depending on the acceptance data) to predict 19 quarters of implementation (2017–2022) in the absence of the intervention. It raises two questions: first, whether a strong negative or positive baseline trend would realistically continue over such an extended period without the intervention taking place and second, whether a different model fit might be better suited to the data. For the latter point, although a linear trend is more simplistic, this was a pragmatic choice which is also robust to outliers, standard in many epidemiological studies, and therefore interpretable to a wide audience. Also, due to the reorganization of districts and the need to merge different datasets to create longitudinal data over such a long period, not all districts could be included in the analysis. The GLM was only based on a relatively small sample size with only 31 time points in total and we did not adjust for any confounding factors. Furthermore, because the evaluation was not intended as an impact evaluation, we did not include a control area. Consequently, time-dependent confounders could not be controlled in the quantitative analysis, limiting the ability to attribute observed changes directly to the intervention. The COVID-19 pandemic also impacted the TB notifications in Ghana [48], which may have influenced the observed trends and outcomes. Studying the trends on non-TB conditions could have given more insights into the wider health systems’ impact of the devices. In general, a wider lung health perspective of the project and the evaluation would have been opportune.

## Conclusion and recommendations

Implementing the ORIO project offered significant potential for enhancing TB case detection, expanding diagnostic capacity, and improving access to digital X-ray services. However, the effectiveness of these systems depends on appropriate utilization, which requires a well-informed understanding of the operational context, governance structures, standardized diagnostic protocols, and clear guidelines. Regular updates to training and procedural documents are essential, especially in response to staff shortage and changes, to ensure continuity and proficiency in usage. Real-time monitoring incorporating essential internet infrastructure should also be prioritized to steer usage of the machines and to optimize the X-ray system’s impact. Together, these measures support digital X-ray technology’s effective and sustainable integration in healthcare facilities, offering a promising path toward improved TB management and a broader diagnostic capability.

## Supporting information

S1 Table Characteristics of patients interviewed. (DOCX)

S2 Table Characteristics of Health Care Workers interviewed. (DOCX)

S1 Checklist Inclusivity in global research. (DOCX)
