# Supplementary material for: A mixed-method evaluation of the impact of introducing 52 digital X-ray systems in Ghanaian health facilities on tuberculosis case finding and quality of care
Source: PLOS Glob Public Health. 2026 Jul 21;6(7):e0006874. doi: 10.1371/journal.pgph.0006874 (PMC13387534; doi:10.1371/journal.pgph.0006874)
Supplement: S1 Table — (DOCX) [file pgph.0006874.s001.docx]

**S1 Table: Characteristics of patients interviewed**

|  | No cough N=19 | | Cough N=11 | | Total N=30 | |
| --- | --- | --- | --- | --- | --- | --- |
| Gender |  |  |  |  |  |  |
| Female | 10 | 53% | 2 | 18% | 12 | 40% |
| Male | 9 | 47% | 9 | 82% | 18 | 60% |
| Age category |  |  |  |  |  |  |
| <35 | 7 | 37% | 2 | 18% | 9 | 30% |
| 35 to 50 | 5 | 26% | 7 | 64% | 12 | 40% |
| >50 | 7 | 37% | 2 | 18% | 9 | 30% |
| Date of having X-ray taken |  |  |  |  |  |  |
| Same day of interview | 9 | 47% | 4 | 36% | 13 | 43% |
| Within the same week | 10 | 53% | 4 | 36% | 14 | 47% |
| More than a month ago | 0 | 0% | 3 | 27% | 3 | 10% |
