# Supplementary material for: A mixed-method evaluation of the impact of introducing 52 digital X-ray systems in Ghanaian health facilities on tuberculosis case finding and quality of care
Source: PLOS Glob Public Health. 2026 Jul 21;6(7):e0006874. doi: 10.1371/journal.pgph.0006874 (PMC13387534; doi:10.1371/journal.pgph.0006874)
Supplement: S2 Table — (DOCX) [file pgph.0006874.s002.docx]

**S2 Table: Characteristics of Health Care Workers interviewed**

|  | Radiographers^*^  N=21 | | TB focal points  N=18 | | Medical doctors N=18 | |
| --- | --- | --- | --- | --- | --- | --- |
| Gender |  |  |  |  |  |  |
| Female | 2 | 10% | 6 | 33% | 3 | 17% |
| Male | 18 | 90% | 12 | 67% | 15 | 83% |
| Age category |  |  |  |  |  |  |
| <35 | 8 | 40% | 9 | 50% | 8 | 44% |
| 35 to 50 | 9 | 45% | 8 | 44% | 8 | 44% |
| >50 | 3 | 15% | 1 | 6% | 2 | 11% |
| For how long have you been working here? | | | |  |  |  |
| Less than 1 year | 0 | 0% | 2 | 11% | 1 | 6% |
| >1 to 3 years | 4 | 20% | 6 | 33% | 8 | 44% |
| >3 to 5 years | 9 | 45% | 3 | 17% | 2 | 11% |
| More than 5 years | 7 | 35% | 7 | 39% | 7 | 39% |

* This includes other staff who are trained to operate the X-ray system
